# Small-angle x-ray scattering investigation of the integration of free fatty acids in polysorbate 20 micelles

**DOI:** 10.1016/j.bpj.2024.03.030

**Published:** 2024-03-27

**Authors:** Jörg Ehrit, Tobias W. Gräwert, Hendrik Göddeke, Petr V. Konarev, Dmitri I. Svergun, Norbert Nagel

## Main text

(Biophysical Journal *122*, 3078–3088; August 8, 2023)

In the originally published version of this article, the following errors appear in Table 1: (1) all values in the fourth column from the left are too large by an order of magnitude, (2) the definition of *N*_*mol*_ in the legend is incorrect, and (3) the value and definition of *I*_*PS20*_ in the legend is incorrect. The corrected version of Table 1 appears below.

**Table:** Table 1. Overall SAXS parameters for PS20 (5 mg/mL), F2 (5 mg/mL), and F4 (5 mg/mL) with no or low amounts of MA (<100 μg/mL) and with high amounts of MA (>500 μg/mL) obtained by GNOM

Sample	*R*_*g*_ (nm)	*D*_*max*_ (nm)	*I*_*0*_ (cm^−1^)	*N*_*mol*_
PS20 (no or <100 μg/mL MA)	3.4 ± 0.1	8.6 ± 0.5	0.0054	34
PS20 (>500 μg/mL MA)	3.5 ± 0.1	8.8 ± 0.5	0.0049	32
F2 (no or <100 μg/mL MA)	2.9 ± 0.1	8.0 ± 0.5	0.0033	22
F2 (>500 μg/mL MA)	3.0 ± 0.1	8.3 ± 0.5	0.0035	23
F4 (no or <100 μg/mL MA)	3.3 ± 0.1	8.4 ± 0.5	0.0051	32
F4 (>500 μg/mL MA)	3.5 ± 0.1	8.7 ± 0.5	0.0055	34

*R*_*g*_, radius of gyration; *D*_*max*_, maximum size; *I*_*0*_, extrapolated zero angle scattering; *N*_*mol*_, approximate number of molecules in the micelle estimated as *I*_*0*_/*I*_*PS20*_, where *I*_*PS20*_ = 0.16 × 10^−3^ cm^−1^ is the computed forward scattering by a size-averaged PS20 molecule in water.

