# Supplementary material for: Small-angle x-ray scattering investigation of the integration of free fatty acids in polysorbate 20 micelles
Source: Biophys J. 2023 Jun 19;122(15):3078–88. doi: 10.1016/j.bpj.2023.06.011 (PMC10432221; doi:10.1016/j.bpj.2023.06.011)
Supplement: Document S1. Figures S1–S4 [file mmc1.pdf]

Biophysical Journal, Volume *122*

Supplemental information

Small-angle x-ray scattering investigation of the integration of free fatty acids in polysorbate 20 micelles

Jörg Ehrit, Tobias W. Gräwert, Hendrik Göddeke, Petr V. Konarev, Dmitri I. Svergun, and Norbert Nagel

Supplementary Material

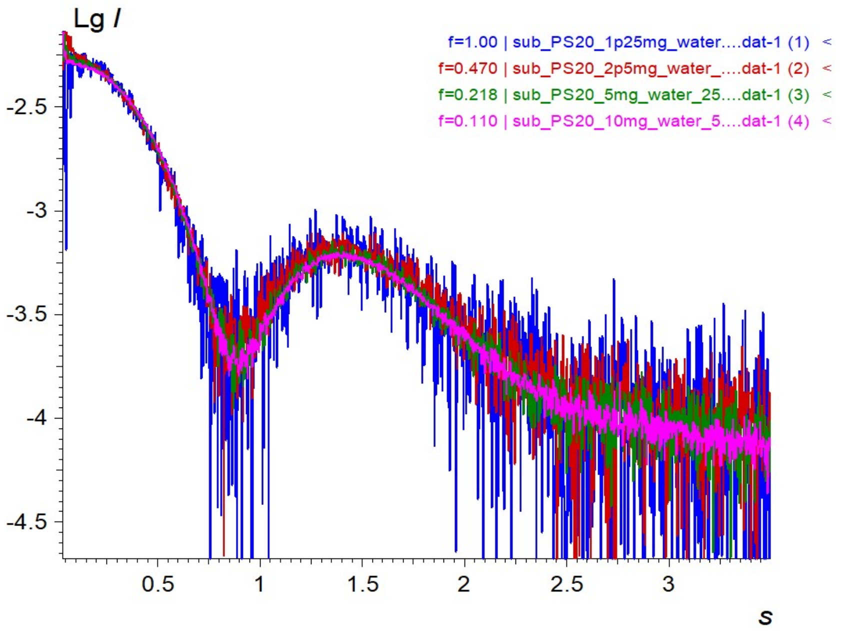

Figure S1. Scattering from PS20 at concentrations 1.25 mg/ml (curve 1), 2.5 mg/ml (curve 2), 5.0 mg/ml (curve 3) and 10.0 mg/ml (curve 4). The s-axis units are nm^{-1}.

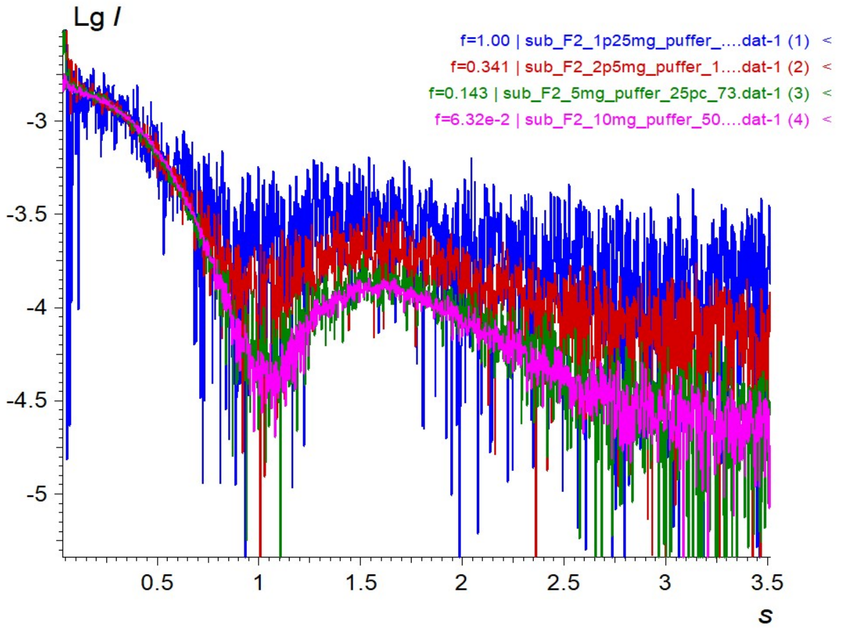

Figure S2. Scattering from fraction F2 at concentrations 1.25 mg/ml (curve 1), 2.5 mg/ml (curve 2), 5.0 mg/ml (curve 3) and 10.0 mg/ml (curve 4). The s-axis units are nm^{-1}.

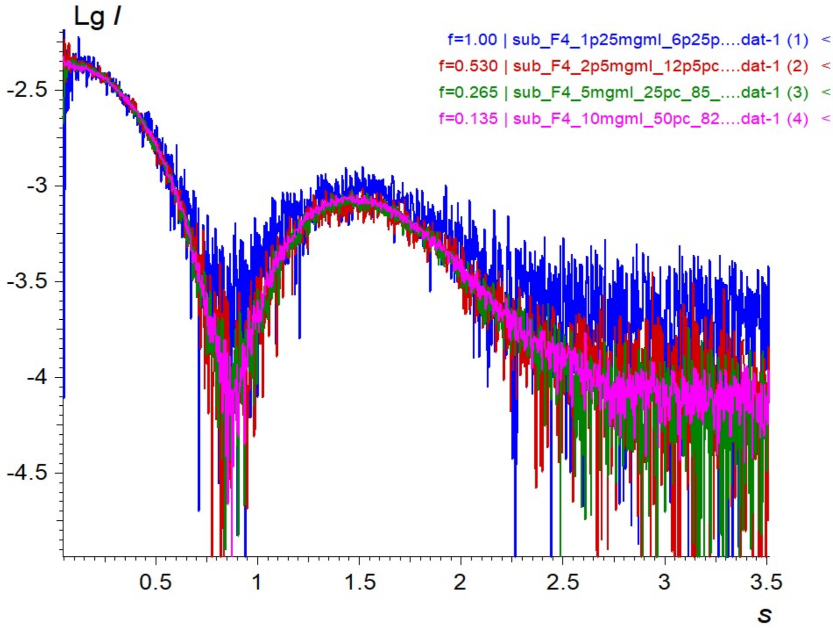

Figure S3. Scattering from fraction F4 at concentrations 1.25 mg/ml (curve 1), 2.5 mg/ml (curve 2), 5.0 mg/ml (curve 3) and 10.0 mg/ml (curve 4). The s-axis units are nm^{-1}.

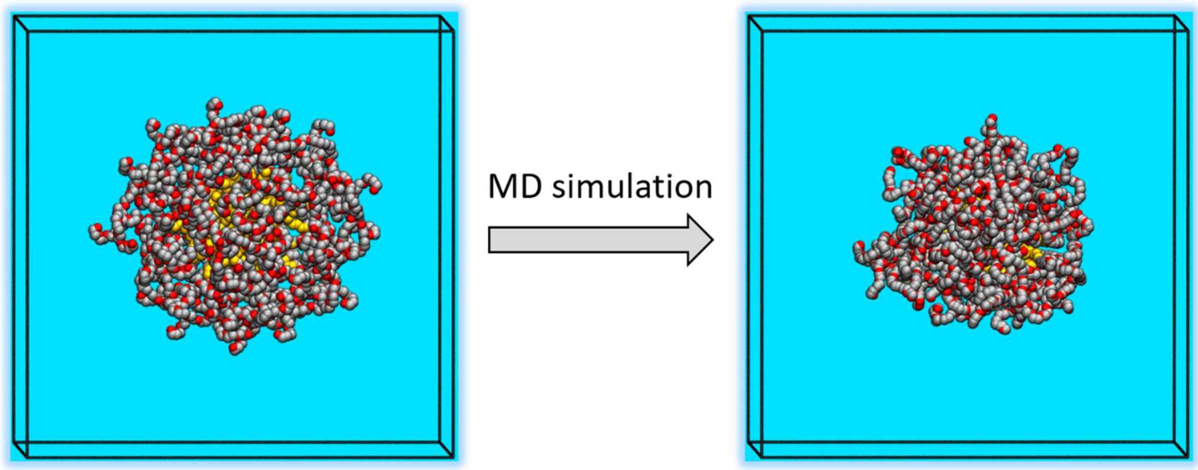

Figure S4. *Snapshots from an MD simulation of a F2 micelle model (generated by OLIGOMER, see. model 6 in Fig. 5) with 35 F2 molecules solvated with water at the start of the simulation (left) and after 300 ns (right). Hydrophobic tails and polar head groups of F2 molecules are shown as yellow spheres and grey/red spheres, respectively. Hydrogens and water molecules are not shown for visual clarity.*

MD models and force field parameters

The compressed zip file "MD-models-and-forcefield-params.zip" includes representative models of the F2 micelle at the start and at the end of the MD simulations (Fig. S4) w/o myristic acid, and at the end of the simulation upon adding myristic acid at a 1:1 mixture (see Fig. 7). All GAFF2 force field parameters of the F2 and myristic acid molecules can be found in "forcefield-params-for-F2-and-MYR.txt". The detailed simulation protocol is outlined in the Materials and Methods section.
