# Supplementary material for: Small-angle x-ray scattering investigation of the integration of free fatty acids in polysorbate 20 micelles
Source: Biophys J. 2023 Jun 19;122(15):3078–88. doi: 10.1016/j.bpj.2023.06.011 (PMC10432221; doi:10.1016/j.bpj.2023.06.011)
Supplement: Document S3. Article plus supporting material [file mmc3.pdf]

Biophysical Journal
Article

Small-angle x-ray scattering investigation of the integration of free fatty acids in polysorbate 20 micelles

Jörg Ehrit,[1] Tobias W. Gräwert,[2] Hendrik Göddeke,[3] Petr V. Konarev,[4] Dmitri I. Svergun,[2,*] and Norbert Nagel[5,*]
[1]Analytical Research and Development, NBE Analytical R&D, AbbVie Deutschland GmbH & Co. KG, Ludwigshafen, Germany; [2]European Molecular Biology Laboratory, Hamburg Unit, Hamburg, Germany; [3]Computational Drug Discovery, Small Molecule Therapeutics & Platform Technologies, AbbVie Deutschland GmbH & Co. KG, Ludwigshafen, Germany; [4]A. V. Shubnikov Institute of Crystallography, Federal Scientific Research Centre "Crystallography and Photonics" of Russian Academy of Sciences, Moscow, Russian Federation; and [5]Analytical Research and Development, Global Technical Centers, AbbVie Deutschland GmbH & Co. KG, Ludwigshafen, Germany

ABSTRACT A critical quality attribute for liquid formulations is the absence of visible particles. Such particles may form upon polysorbate hydrolysis resulting in release of free fatty acids into solution followed by precipitation. Strategies to avoid this effect are of major interest for the pharmaceutical industry. In this context, we investigated the structural organization of polysorbate micelles alone and upon addition of the fatty acid myristic acid (MA) by small-angle x-ray scattering. Two complementary approaches using a model of polydisperse core-shell ellipsoidal micelles and an ensemble of quasiatomistic micelle structures gave consistent results well describing the experimental data. The small-angle x-ray scattering data reveal polydisperse mixtures of ellipsoidal micelles containing about 22–35 molecules per micelle. The addition of MA at concentrations up to 100 μg/mL reveals only marginal effects on the scattering data. At the same time, addition of high amounts of MA (>500 μg/mL) increases the average sizes of the micelles indicating that MA penetrates into the surfactant micelles. These results together with molecular modeling shed light on the polysorbate contribution to fatty acid solubilization preventing or delaying fatty acid particle formation.

SIGNIFICANCE Polysorbate-containing formulations of biologics may occasionally release free fatty acids upon polysorbate degradation. These free fatty acids may precipitate and form particles, which is undesirable for pharmaceutical formulations for subcutaneous injection. As polysorbate micelles should have the potential to incorporate free fatty acids and thus prevent their precipitation, a better understanding of the interaction of free fatty acids with polysorbate micelles should help to develop more robust pharmaceutical formulations of biologics. Here, we present molecular modeling and experimental results that provide detailed information on interaction of free fatty acids with polysorbate micelles.

INTRODUCTION

Pharmaceutical drugs do not only contain an active pharmaceutical ingredient (API); in general, the API is assisted by several excipients and all together comprise the formulated drug product. A very important aim of the formulation is to keep the API intact, active, and available over the shelf-life. Temperature, pH, oxidative, UV, agitation, and shear stress are the major factors APIs need to be protected against in biologics formulations (1–3). Surface active agents, short surfactants, are a molecule class widely used in pharmaceutical formulations to ensure the product quality (4). These molecules are amphiphilic with hydrophilic and hydrophobic regions and therefore locate energetically preferred at phase boundaries between polar and nonpolar phases. Examples of such boundaries are the liquid-air or liquid-container interfaces in formulations. The protective mechanism is well studied, and the API stabilization is mostly linked to a competitive adsorption of a surfactant to the hydrophobic interfaces and a resulting displacement of the API (1,2,5–9). Proteins are the APIs in biologics and surfactants are able to shield the hydrophobic surfaces at the boundaries of the

Submitted February 24, 2023, and accepted for publication June 15, 2023.
*Correspondence: svergun@embl-hamburg.de or norbert.nagel@abbvie.com
Tobias W. Gräwert's and Dmitri I. Svergun's present address is BioSAXS GmbH, Hamburg, Germany.
Editor: Heiko Heerklotz.
https://doi.org/10.1016/j.bpj.2023.06.011
© 2023 Biophysical Society.
This is an open access article under the CC BY license (http://creativecommons.org/licenses/by/4.0/).

protein solution thereby preventing adsorption, misfolding of the proteins, and aggregation at these interfaces (10–12).

The formation of surfactant micelles is a further possibility to reduce the free energy of amphiphilic surfactants in polar solutions and occurs above a certain surfactant concentration, the critical micelle concentration (13–16). The surfactant molecules are oriented with the hydrophilic regions directed to the outside of the micelle while the hydrophobic regions cluster in the micellar core. These structures can differ in size, shape, aggregation number (number of molecules per micelle), and stability. The micellar appearance is also dependent on the type of surfactant, concentration, and environmental conditions (e.g., pH and temperature). The resulting structures can vary from small spherical, via elongated cylindrical or large flat lamellar micelles to vesicles with a bilayer (17).

Polysorbates (PSs), also known as Tweens, are very common dispersants and are the most relevant surfactants in therapeutic biologics. Ninety-six percent of all commercial biologics' formulations include either PS20 or PS80 (4,18). Both PSs consist of a sorbitan core, substituted with on average 20 polyoxyethylene (POE) subunits. This hydrophilic part of the molecule is esterified with a hydrophobic fatty acid. The used fatty acid mixture defines the difference between PS20 and PS80. Oleic acid (C18:1) is the predominant fatty acid in PS80 and lauric acid (C12:0) in PS20 (Fig. 1). PS has no clearly defined structure being a mixture of many different structures. Already the condensation of sorbitol to sorbitan can result in isosorbide. These core structures can be substituted with many different POE patterns and one or several of different fatty acids. All this ends up in more than 1500 possible structures for each PS (19). PS micelles typically contain a mixture of different structures and the composition varies with PS concentration (20). PSs used in pharmaceutical formulations are regulated via compendial requirements and the batch-to-batch consistency is highly controlled by raw material testing.

Numerous publications are devoted to issues related to PS degradation in biologics (2,21–30). The most frequently occurring issues are loss of functional PS, the appearance of particles or enhanced oxidation. These phenomena are related to PS degradation, which is described to occur via two pathways, a hydrolytic and an oxidative one. The oxidative pathway is based on a radical-driven mechanism and is more prominent for PS80 than for PS20. The omnidirectional oxidative degradation is also described to foster protein oxidation (31) and could therefore influence the quality of biologics. However, this pathway is not the focus of this publication. The second pathway, PS hydrolysis, results in an ester cleavage that can be caused either by extreme pH or by hydrolytic enzymes. The pH is always moderate in biologics and is therefore not of high relevance. Consequently, together with the API, copurified host cell proteins with esterase activity are the more probable root cause for hydrolytic PS degradation. This hydrolytic pathway results in a release of fatty acids from PS into the protein solution. Most of the released fatty acids are poorly soluble and tend to aggregate into particles (24,26). Such particles could be a critical quality attribute of biologics.

Many parameters influence the fatty acid solubility. Important factors in biopharmaceuticals are the type of fatty acid, pH, silicone oil concentration, and the PS concentration (32–34). The chain length and saturation of fatty acids influence the solubility and the solid state. Longer fatty acids are less soluble in aqueous solutions than shorter ones. Furthermore, the melting point is influenced by the alkyl chain length and saturation. The longer but unsaturated oleic acid (C18:1) is liquid at room temperature while a shorter but saturated lauric acid (C12:0) is solid. The pH influences the charge state of the fatty acid and therefore also the solubility in aqueous solutions. In general, all formulation components can influence the fatty acid solubility, from silicone oil via the API to surfactants. Well studied is the positive effect of PSs on the solubility of free fatty acids (33,35), but it remains unknown what happens on the molecular level in these solutions. Our aim was to shed light on the question, whether free fatty acids released from PS20 solutions can be incorporated into PS20 micelles and whether micelles of different PS20 fractions behave differently with respect to fatty acid incorporation. Investigations were performed on PS20, the POE sorbitan monolaurate fraction (F2) and a fraction of POE sorbitan esterified with more than one lauric acid (F4). Myristic acid (MA) (C14:0) was used as model free fatty acid in the experiments. MA is the second most abundant fatty acid of PS20 and less soluble than the most abundant fatty acid lauric acid. Considering these properties, we expect MA to have a higher relevance for the occurrence of particles in biologics liquid formulations.

Different experimental techniques were used in the past to investigate the surfactant behavior and micellar structure in solution, from tensiometric methods via dynamic light scattering to small-angle scattering of x-rays (SAXS) and neutrons. Small-angle scattering yields structural information on the nanometer scale being suitable for common surfactant micelles. Here, we employed SAXS using high-brilliance synchrotron radiation providing accurate experimental data on low-concentration solutes. SAXS uses the elastic scattering behavior of x-rays traveling through material and interacting with the electrons. The scattering data after subtraction of the solvent contribution contain information about size, shape, and electron density distribution of the investigated structure. To get further insights of PS20 structure, molecular dynamics (MD) simulations have been used to study PS20 micelles and their interaction with small molecules (36). In this work, we also used MD simulations to model the interactions between the PS20 micelles and MA, and shed some light on how the MA molecules migrate into the PS20 micelles.

This study demonstrates experimentally and by modeling that free fatty acids can be incorporated into the PS micelles. This information is important for the mechanistic understanding of how does PS contribute to the solubilization of fatty acids.

MATERIALS AND METHODS

Materials

Polysorbate NF, Multi-Compendial, from J.T. Baker was used for the experiments with whole PS20. POE sorbitan monolaurate fraction (F2) and the higher-order esters fraction (F4) of lauric acids with POE sorbitan were purified and provided by Croda (East Yorkshire, UK). PS and the PS fractions were dissolved in Milli-Q water. MA (Merck, Darmstadt, Germany) was dissolved in methanol (Avantor, Luxembourg). *n*-Dodecyl-β-maltoside (DDM) (Sigma-Aldrich, Darmstadt, Germany) was dissolved in Milli-Q water.

Sample preparation

PS (PS20, F2, F4) stock solutions were diluted to useful intermediate concentrations with ultrapure Milli-Q water and stirred for 10 min or vortexed for 5 min. A stock solution of MA in methanol was diluted to useful intermediate concentrations with methanol. A titration series of MA from 0 to 2000 μg/mL in 5 or 1 mg/mL PS was then pipetted, where the methanol content was kept at 4% (v/v). The sample system was kept without any buffer or further formulation components to reduce potential sources of errors when subtracting placebo from verum. Samples were vortexed for 1 min and then incubated for 16 h at room temperature. For higher MA concentrations, a white precipitate was observed, and the supernatant was carefully transferred into a fresh vessel for measurements. For DDM measurements, 7.3 mg DDM was dissolved in a final volume of 730 μL Milli-Q water immediately before SAXS measurements.

SAXS measurements

SAXS data from PS PS20, F4, F2, and DDM samples were collected at the EMBL SAXS beamline P12 at PETRA III, DESY, Hamburg, Germany (37). The scattering intensity $I(s)$ was measured as a function of momentum transfer $s = 4\pi sin\Theta/\lambda$, where 2Θ is the scattering angle and λ the x-ray wavelength. The PS samples were measured in an aqueous solution of 4% methanol ("buffer"). The titration against MA was conducted at detergent concentrations of 1 and 5 mg/mL, with addition of MA from 0 to 2000 μg/mL. For the concentration effect analysis on PS samples, a serial dilution (10, 5, 2.5, and 1.25 mg/mL) was conducted. DDM was measured in Milli-Q water at detergent concentrations 10, 5, 2.5, and 1.25 mg/mL, with Milli-Q water measured as blank. SAXS data were collected at 20°C using a batch mode setup with automated sample delivery (38). The beamline was calibrated for angular axis using silver behenate powder. The scattering data were collected by a two-dimensional PILATUS-6M detector with the sample to detector distance 4 m and the x-ray energy 8 keV. The scattering data underwent standard automated radial averaging and buffer subtraction procedures (39). Samples and the corresponding matching solvents were measured under continuous flow to avoid radiation damage. For each specimen, 40 frames with exposure time 0.1 s were collected and the successive frames were analyzed for possible radiation damage using the pipeline SASFLOW (40). The frames showing potential radiation damage effects (i.e., statistically different from the first frame) were not included in the average. The capillary was washed after each sample and the measurements of all solutes were repeated 6–10 times and independent repetitions were averaged to reduce the noise (39). The overall particle parameters were assessed by PRIMUS (41). The distance distribution functions were computed by an indirect Fourier transformation using GNOM (42).

Modeling using a system of polydisperse core-shell ellipsoidal micelles

SAXS data from PS20, F2, and F4 were approximated by a system of polydisperse core-shell ellipsoidal micelles using the program BILMIX (43). This program was originally developed for the analysis of spherical or ellipsoidal unilamellar lipid vesicles, but it has the capacity for modeling of ellipsoidal micelles as well. The following parameters of the micelle system are optimized by BILMIX: semiaxes of the ellipsoid of revolution (a,a,b), polydispersity of the ellipsoid dR_{ell}, thickness of the ellipsoidal shell t, electron densities of the core (ρ_{in}) and shell (ρ_{out}) parts of the micelle. The scheme of a core-shell ellipsoidal model of a micelle is shown in Fig. 2.

Modeling using quasiatomistic models of micelles

In an alternative approach, we have used a number of quasiatomistic models of micelles generated by the program ELLMIC, a modified version of ELLLIP (44). ELLMIC uses a monomeric PS20 molecule as an individual building block. For this, quasihomogeneous angular grids with a user-specified number of directions based on Fibonacci numbers sequence (1, 2, 3, 5, 8, 13, 21, 34 …) are generated. Then the monomeric lipids are aligned with respect to these axes to build an ellipsoid with the specified semiaxes.

A number of prolate and oblate ellipsoids with varying sizes were generated based on the modeling results of the previous section. From the experimental zero angle scattering (and also from the earlier results), the micelles were found to contain between about 20 and 35 surfactant molecules. Two types of micelles were thus generated containing 22 and 35 monomeric surfactants. These micelles included model 1 (ellipsoid with semiaxes a = 4.5 nm, b = 3.4 nm, c = 3.3 nm), model 2 (a = 4.5 nm, b = 4.5 nm, c = 3.5 nm), model 3 (a = 5.0 nm, b = 3.5 nm, c = 3.5 nm), model 4 (a = 5.0 nm, b = 4.5 nm, c = 4.0 nm), model 5 (a = 4.0 nm, b = 3.0 nm, c = 3.0 nm), and model 6 (a = 5.0 nm, b = 4.0 nm, c = 4.0 nm). Besides, 15 different conformations of monomeric surfactant molecules were used as individual building blocks (with different tail lengths). In total, an ensemble of 180 quasiatomistic models was created.

The theoretical scattering intensities from the generated micelles were calculated using the program CRYSOL (45). To account for the polydispersity of the micelles, the data were fitted by linear combinations of the calculated theoretical intensities from multiple models using the program OLIGOMER (41). Given the intensities from the components (surfactant micelles) OLIGOMER finds their volume fractions by solving a system of linear equations using the algorithm of nonnegative least-squares to minimize the discrepancy between the experimental and calculated scattering curves. The models yielding systematically nonzero volume fractions in the OLIGOMER fitting were selected.

MD simulations

All simulations were carried out with the GROMACS program package, version 2020.4 (46). The structure of F2 (C12) was modeled with equal polyethylene glycol chain length (w = x = y = z = 5, see Fig. 1), which is a major simplification as PS20 is a mixture of roughly 1500 different structures (19). The F2 micelle with 35 F2 molecules built by ELLMIC (Fig. 5 *b*, *top right*) was centered in a periodic simulation box (x, y, z dimensions were ~11.8, 10.9, and 10.0 nm) solvated with ca. 40,000 TIP3P water molecules. The system size was ca. 125,000 atoms. After energy minimization (1000 steps steepest descent), 2 × 300 ns MD simulations were carried out in the NpT ensemble and the first 10 ns were discarded as equilibration. The final structure of the first simulation was subjected to additional simulations with MA at three different F2:MA mixtures (ca. 2:1, 1:1, 1:2); 20, 35, and 70 MA molecules were randomly placed in the solvent of the simulation box and overlapping water molecules were removed. Na^+ ions were used to neutralize the negative charges of the MA molecules. After energy

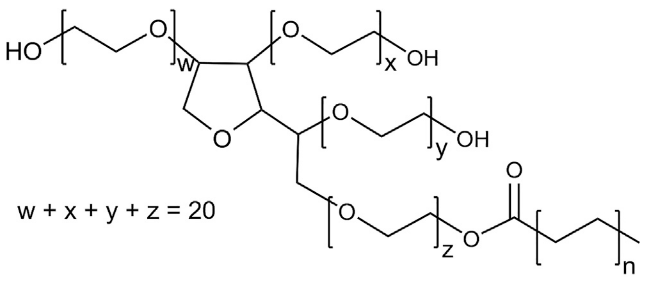

FIGURE 1 Polysorbate average structure. The sum of w, x, y, and z, the number of POE substituents, is on average 20 and *n* can vary from 2 to 8.

minimization (1000 steps steepest descent), one 300 ns MD simulation was carried out for every F2:MA mixture and the first 10 ns were discarded as equilibration. The general AMBER force field (GAFF) version 2.1 (47) with AM1-BCC partial charges was used for the F2 and MA molecules. Long-range electrostatic interactions were treated with the particle mesh Ewald method (48) with a grid spacing of 0.16 nm. Short-range van der Waals interactions were described with a Lennard-Jones 6–12 potential that was cut off at 1.0 nm. This cutoff was also used for the division between short- and long-range Coulomb interactions. The SETTLE algorithm (49) was used to constrain the bonds and angles of the water molecules, and LINCS (50) was used to constrain all bonds with H-atoms. Together with hydrogen mass repartitioning ($m = 3.024$ u) for the F2 and MA hydrogens (51), allowed for an integration timestep of 4 fs using the leap-frog integrator. The temperature was kept constant at 300 K by coupling to a velocity rescaling thermostat with a coupling time constant of 0.4 ps (52). A Berendsen barostat was used for isotropic pressure coupling at 1 bar with a time constant of 2.0 ps and compressibility of 4.5×10^{-5} bar^{-1}. The accumulated simulation time was 1500 ns with a throughput of 200 ns/day (Intel Xeon Gold 6148/NVIDIA RTX 6000).

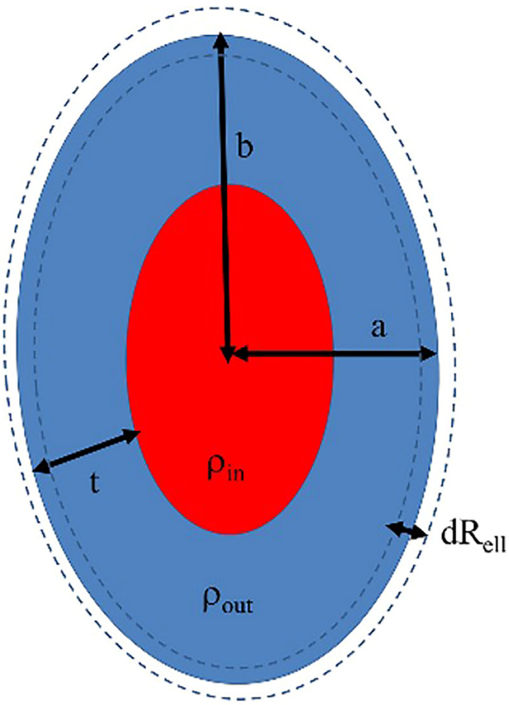

FIGURE 2 Schematic representation of a polydisperse core-shell ellipsoidal micelle. The parameters describing the model are the following: semiaxes of the ellipsoid of revolution (*a,a,b*), polydispersity of the ellipsoid dR_{ell}, thickness of the ellipsoidal shell *t*, electron densities (contrasts) of the core (ρ_{in}) and shell (ρ_{out}) parts of the micelle. To see this figure in color, go online.

RESULTS

To assess the concentration effects for PS20 and fractions F2 and F4, SAXS data were recorded at 1.25, 2.5, 5.0, and 10.0 mg/mL PS without addition of MA (Figs. S1–S3). The measurement was performed in water without further components to avoid interference. Here, the scattering data from F4 and PS20 demonstrate only minor concentration-related difference in the measured concentration range. This observation suggests that the overall shapes and organization of the micelles are essentially concentration independent in the investigated range for PS20 and the F4 fraction. For the F2 fraction, the two lowest concentrations, 1.25 and 2.5 mg/mL, reveal an elevated intensity at higher scattering angles (starting from about 0.5 [1/nm]) compared with the two data sets taken at higher concentrations (5 and 10 mg/mL). This result may be partially explained by an equilibrium between the F2 micelles and monomers. However, as the R_g value does not seem to decrease with concentration, a mechanism suggested by Knoch et al. may better explain the observed effect (20). Here, at low concentrations, the initial micelles could be built from the surfactant species with the lowest individual cmc, which are more sparsely packed but yield micelles of the same size. Starting from c = 5 mg/mL, no further concentration difference is observed and the scattering from F2 at 5 mg/mL agrees well with that from F2 at 10 mg/mL.

The presented data indicate that the concentration of 5 mg/mL is optimal to study the organization of PS micelles (sufficiently high to prevent dissociation and sufficiently low to avoid interparticle interference effects). The overall structural parameters derived from the SAXS data without addition of MA are summarized in Table 1. The SAXS results for PS20 and its fractions are in a good agreement with the small-angle neutron scattering data recently published for similar PS20 samples (53). One can see a slight increase of the radius of gyration R_g and the maximum particle size D_{max} upon adding high amounts of MA (Fig. 3). The number

TABLE 1 Overall SAXS parameters for PS20 (5 mg/mL), F2 (5 mg/mL), and F4 (5 mg/mL) with no or low amounts of MA (<100 μg/mL) and with high amounts of MA (>500 μg/mL) obtained by GNOM

Sample	R_g (nm)	D_{max} (nm)	I_0 (cm^{-1})	N_{mol}
PS20 (no or <100 μg/mL MA)	3.4 ± 0.1	8.6 ± 0.5	0.054	34
PS20 (>500 μg/mL MA)	3.5 ± 0.1	8.8 ± 0.5	0.049	32
F2 (no or <100 μg/mL MA)	2.9 ± 0.1	8.0 ± 0.5	0.033	22
F2 (>500 μg/mL MA)	3.0 ± 0.1	8.3 ± 0.5	0.035	23
F4 (no or <100 μg/mL MA)	3.3 ± 0.1	8.4 ± 0.5	0.051	32
F4 (>500 μg/mL MA)	3.5 ± 0.1	8.7 ± 0.5	0.055	34

R_g, radius of gyration; D_{max}, maximum size; I_0, extrapolated zero angle scattering; N_{mol}, average number of molecules in the micelle estimated as I_0/I_{PS20}, where $I_{PS20} = 0.227 \times 10^{-3}$ cm^{-1} is the computed forward scattering by a single PS20 molecule in water.

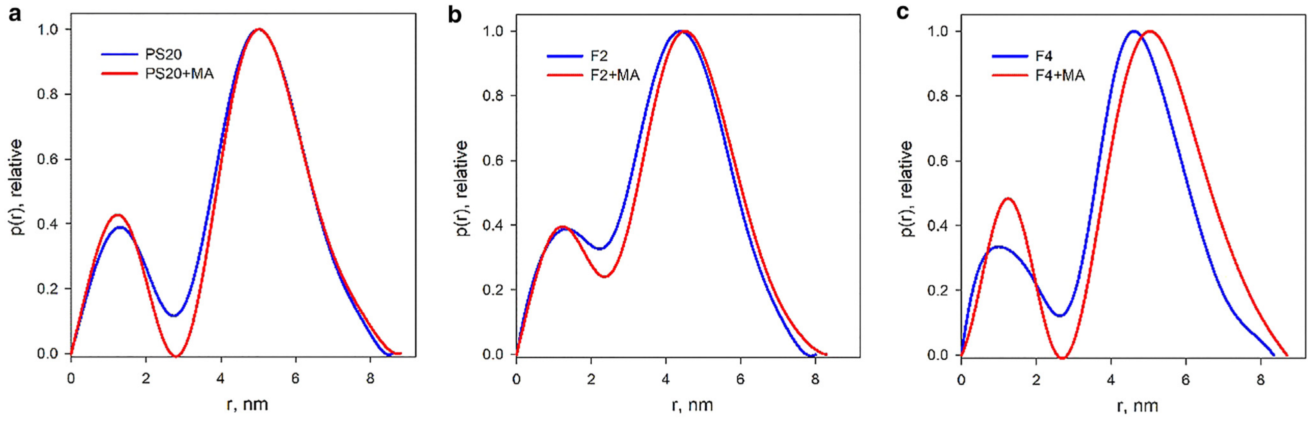

FIGURE 3 Distance distribution functions for PS20 (*a*), F2 (*b*), and F4 (*c*) micelles at 5 mg/mL: blue curves, with no MA; red curves, with high amount of MA ("+MA") (>500 μg/mL). To see this figure in color, go online.

of surfactant molecules in the micelles can be estimated between 22 and 35 according to the extrapolated zero angle scattering I_0 values as obtained from GNOM analysis.

The modeling results in terms of polydisperse core-shell ellipsoidal micelles are presented in Tables 2, 3, 4, and 5. The experimental SAXS data and their best fits by BILMIX are shown in Fig. 4. As seen from Table 1, R_g and D_{max} and also the number of molecules per surfactant micelles are noticeably smaller for F2 micelles compared with those of PS20 and F4 micelles. PS20 and F4 micelles appear to be rather similar to each other, F4 being just slightly smaller and (from the BILMIX fitting in Table 2) less polydisperse than PS20. For all fits, the obtained shell density is positive (lipid heads) and the core density is negative (lipid tails). In an unconstrained search of the best fits, BILMIX typically selects oblate ellipsoids; however, a system of prolate ellipsoids can also reasonably well describe the data, providing just slightly worse fits to the data. The addition of MA at concentrations up to 100 μg/mL reveals only marginal effects on the scattering data (the changes are within the error estimates of the micelle sizes). At the same time, for all samples, addition of higher amounts of MA (>500 μg/mL) noticeably increases the average sizes of the micelles (see Tables 3, 4, and 5).

In the BILMIX modeling, the contrasts of the core and shell (ρ_{in} and ρ_{out}) are evaluated on a relative scale by the minimization program. Interestingly, however, the ratio of the obtained contrasts of the two components is about −0.55, which is expected for the theoretical ratio of the contrast of the lipid tails (−0.036 e/A^3) to that of the polar heads (0.059 e/A^3). This ratio, determined by BILMIX without information on the contrasts, lends further support to the models.

In an alternative approach, quasiatomistic models of micelles of different sizes were generated and tested (see materials and methods). With this approach it was possible to fit the data well by linear combinations of the calculated theoretical intensities of micelles as shown in Fig. 5 *a*. The results of the modeling are presented in Table 6. In all cases, the data could not be satisfactorily fitted by any single model and from two to four different models were selected upon screening. This finding is not surprising given the expected polydispersity of the micelles. The selected models can be separated into two groups: group 1, "large" micelles (with semiaxes between 4.5 and 5.0 nm, 3.5 and 4.0 nm, 3.5 and 4.0 nm, respectively) and group 2, "small" micelles (with semiaxes of 4.0, 3.0, and 3.0 nm). Typical models selected by OLIGOMER are displayed in Fig. 5 *b*.

TABLE 2 Structural parameters of polydisperse core-shell ellipsoidal micelles for PS20 (5 mg/mL), F2 (5 mg/mL), and F4 (5 mg/mL) with no or low amounts of MA (<100 μg/mL) and with high amounts of MA (>500 μg/mL) obtained by BILMIX

Sample	Ellipsoid semiaxis, *a* (nm)	Ellipsoid semiaxis, *b* (nm)	Dispersion of ellipsoid, dR_{ell} (nm)	Thickness of ellipsoidal shell, *t* (nm)	Core density contrast, r_{in} (e/Å^3)	Shell density contrast, r_{out} (e/Å^3)
PS20 (no or < 100 μg/mL MA)	3.68 ± 0.07	2.46 ± 0.07	0.37 ± 0.07	0.88 ± 0.07	−0.035 ± 0.002	0.060 ± 0.004
PS20 (>500 μg/mL MA)	3.70 ± 0.07	2.45 ± 0.07	0.35 ± 0.05	0.97 ± 0.09	−0.039 ± 0.003	0.053 ± 0.002
F2 (no or <100 μg/mL MA)	3.35 ± 0.05	2.11 ± 0.11	0.45 ± 0.09	0.80 ± 0.07	−0.028 ± 0.001	0.055 ± 0.002
F2 (>500 μg/mL MA)	3.43 ± 0.05	2.18 ± 0.09	0.41 ± 0.09	0.80 ± 0.05	−0.031 ± 0.002	0.054 ± 0.002
F4 (no or <100 μg/mL MA)	3.46 ± 0.06	2.19 ± 0.09	0.31 ± 0.07	0.80 ± 0.07	−0.043 ± 0.003	0.057 ± 0.003
F4 (>500 μg/mL MA)	3.65 ± 0.09	2.52 ± 0.07	0.25 ± 0.05	0.80 ± 0.08	−0.031 ± 0.002	0.056 ± 0.003

[a]The error estimates for the model parameters in Tables 2, 3, 4, and 5 were assessed by averaging the variations of the restored values as functions of the fitting interval around the data range corresponding to the number of Shannon channels in the fit ($N_{shannon} = 6$).

TABLE 3 Structural parameters of polydisperse core-shell ellipsoidal micelles for PS20 in the absence of MA and with addition of MA (500, 1000, 1500, and 2000 μg/mL) obtained by BILMIX

PS20 (5 mg/mL)	Ellipsoid semiaxis, a (nm)	Ellipsoid semiaxis, b (nm)	Dispersion of ellipsoid, dR_{ell} (nm)	Thickness of ellipsoid shell, t (nm)	Core density contrast, r_{in} (e/Å^3)	Shell density contrast, r_{out} (e/Å^3)
0 MA	3.68 ± 0.05	2.46 ± 0.07	0.36 ± 0.05	0.82 ± 0.05	−0.031 ± 0.002	0.064 ± 0.003
500 MA	3.70 ± 0.07	2.49 ± 0.06	0.35 ± 0.07	0.91 ± 0.09	−0.035 ± 0.002	0.057 ± 0.002
1000 MA	3.75 ± 0.09	2.52 ± 0.05	0.36 ± 0.05	0.91 ± 0.07	−0.036 ± 0.001	0.058 ± 0.002
1500 MA	3.75 ± 0.07	2.50 ± 0.07	0.37 ± 0.06	0.80 ± 0.09	−0.036 ± 0.002	0.058 ± 0.003
2000 MA	3.68 ± 0.05	2.46 ± 0.07	0.36 ± 0.05	0.82 ± 0.05	−0.033 ± 0.003	0.063 ± 0.004

As seen from Table 6, for all samples large micelles are present in solution, whereas small micelles are found only for F2 samples ("models 5"). This is consistent with the results from BILMIX analyses showing that the average sizes of micelles are smaller for F2 compared with PS20 and F4. The volume fractions of the components are changing with the addition of excess MA. One has also to note that, while F2 data can be fitted by a mixture of micelles containing from 22 to 35 molecules, for PS20 and F4 data only the micelles containing 35 molecules are required.

As a caveat, one should interpret the above results literally as a coexistence of a few separated and distinct structures. Instead, the selected models are tentative representatives of otherwise continuous ensembles. The different structures just represent typical visualizations emerging from an average of continuous and possibly broad variations of micelle shapes over the ensemble and time.

To further verify the results regarding the composition of the micelles, the obtained data were compared with the scattering from DDM micelles. The latter are a well characterized system with known composition and *MW* of about 100 kDa (9). The measured scattering from DDM micelles yielded $R_g = 3.1$ nm, in a good agreement with the data published by Gabel et al. (54). Fig. 6 presents the experimental scattering data from PS20 and DDM on the absolute scale and the best fitting computed scattering curves from a single conformation of the two micelles. For PS20, an ellipsoidal micelle has semiaxes of 4.5, 4.5, and 3.0 nm containing 22 molecules ($MW = 28$ kDa); for DDM, the micelle has semiaxes of 4.0, 3.0, and 3.0 nm containing 174 molecules ($MW = 89$ kDa). Importantly, the scattering from the models was computed without adjusting any fitting parameters; the data were only multiplied by factors slightly exceeding unity (1.05 for PS20 and 1.20 for DDM). The computed curves yield good agreement with the experimental data at small angles, and not only on the curve geometry but also on the number of molecules (i.e., on the absolute scale). One should further mention here the results by Ivanović et al., who utilized the data published by Gabel et al. to analyze the DDM micelles (54,55). The models reported by Ivanović et al. accounting for significant contributions from the bound waters had the overall sizes agreeing with our results. The authors also found that polydisperse ellipsoidal representations of the micellar shapes were in a quantitative good agreement with a multiple-replica ensemble refinement from MD simulations.

The above comparison further confirms that the *MW* of PS20 micelles is around 30 kDa, whereas that of DDM micelles is about 100 kDa, agreeing well with published data (9). Interestingly, despite the much lower *MW* of PS20 micelles compared with DDM, the R_g of PS20 micelles (3.7 nm) is larger than that of DDM micelles (3.1 nm). This points to a rather different organization of the scattering matter in PS20 micelles compared with DDM due to the positioning of the bulkier headgroups of PS20 at the periphery of the micelle.

Furthermore, all-atom MD simulations were performed to investigate qualitatively how the MA molecules interact with PS and if they migrate into the micelles. Due to the computational complexity of the simulation, we decided to study only the OLIGOMER-selected model composed of PS molecules belonging to the F2 fraction (lauric acid monoester, formula in Fig. 1, with $w = x = y = z = 5$) with 35 molecules (model 6; 1-c18). First, 2 × 300 ns MD simulations of one fully solvated lauric acid monoester micelle per simulation box w/o MA were carried out to equilibrate the initial model. In the course of the simulations, the micelles became more compact and spherical,

TABLE 4 Structural parameters of polydisperse core-shell ellipsoidal micelles for F2 in the absence of MA and with addition of MA (1.5, 100, 500, and 1500 μg/mL) obtained by BILMIX

F2 (5 mg/mL)	Ellipsoid semiaxis, a (nm)	Ellipsoid semiaxis, b (nm)	Dispersion of ellipsoid, dR_{ell} (nm)	Thickness of ellipsoid shell, t (nm)	Core density contrast, r_{in} (e/Å^3)	Shell density contrast, r_{out} (e/Å^3)
0 MA	3.33 ± 0.05	2.09 ± 0.09	0.45 ± 0.08	0.80 ± 0.05	−0.031 ± 0.002	0.062 ± 0.003
1.5 MA	3.35 ± 0.05	2.13 ± 0.08	0.44 ± 0.09	0.80 ± 0.06	−0.032 ± 0.003	0.063 ± 0.004
100 MA	3.36 ± 0.07	2.15 ± 0.07	0.41 ± 0.07	0.80 ± 0.07	−0.031 ± 0.002	0.062 ± 0.003
500 MA	3.41 ± 0.06	2.19 ± 0.07	0.40 ± 0.09	0.80 ± 0.05	−0.032 ± 0.002	0.062 ± 0.002
1500 MA	3.41 ± 0.07	2.18 ± 0.05	0.40 ± 0.05	0.80 ± 0.06	−0.032 ± 0.003	0.062 ± 0.003

TABLE 5 Structural parameters of polydisperse core-shell ellipsoidal micelles for F4 in the absence of MA and with addition of MA (1.5, 100, 500, and 1500 μg/mL) obtained by BILMIX

F4 (5 mg/mL)	Ellipsoid semiaxis, a (nm)	Ellipsoid semiaxis, b (nm)	Dispersion of ellipsoid, dR_{ell} (nm)	Thickness of ellipsoid shell, t (nm)	Core density contrast, r_{in} (e/Å^3)	Shell density contrast, r_{out} (e/Å^3)
0 MA	3.46 ± 0.11	2.18 ± 0.12	0.30 ± 0.07	0.80 ± 0.05	−0.036 ± 0.002	0.057 ± 0.002
1.5 MA	3.57 ± 0.09	2.42 ± 0.11	0.22 ± 0.09	1.00 ± 0.07	−0.039 ± 0.003	0.057 ± 0.002
100 MA	3.55 ± 0.11	2.37 ± 0.09	0.23 ± 0.07	1.00 ± 0.06	−0.044 ± 0.003	0.056 ± 0.003
500 MA	3.60 ± 0.07	2.50 ± 0.09	0.17 ± 0.09	1.00 ± 0.09	−0.041 ± 0.002	0.056 ± 0.003
1500 MA	3.72 ± 0.08	2.18 ± 0.11	0.21 ± 0.08	1.00 ± 0.07	−0.044 ± 0.002	0.056 ± 0.002

and their size significantly decreased by around 10–15% compared with the starting models (for more information see supporting material). However, given the polydispersity of the micelles and the fact that F2 and the other fractions are heterogeneous mixtures of multiesters, this is not unexpected. In addition, the size and shape of the resulting micelles are comparable with those found by Lapelosa et al. (36). The final structure of these two MD simulations were used as a starting structure for subsequent MD simulations at three different lauric acid monoester/MA molecular mixtures (1:2, 1:1, and 2:1). The experimental concentrations of 5 mg/mL for the monoester fraction F2 and 1 mg/mL for MA correspond to a molecular F2/MA ratio of approximately 1:1. All simulations show that the MA molecules indeed migrate into the core of the lauric acid monoester micelles leading to an increased size of the micelles by around 5–10% depending on the mixture, which is in line with the SAXS data. The hydrophobic tails of MA and the lauric acid monoester stack with each other and the carboxylic acid of MA points toward the hydrophilic outer shell of the micelles (Fig. 7, *inset*). Although these results are very intriguing, our goal was not to get a quantitative comparison

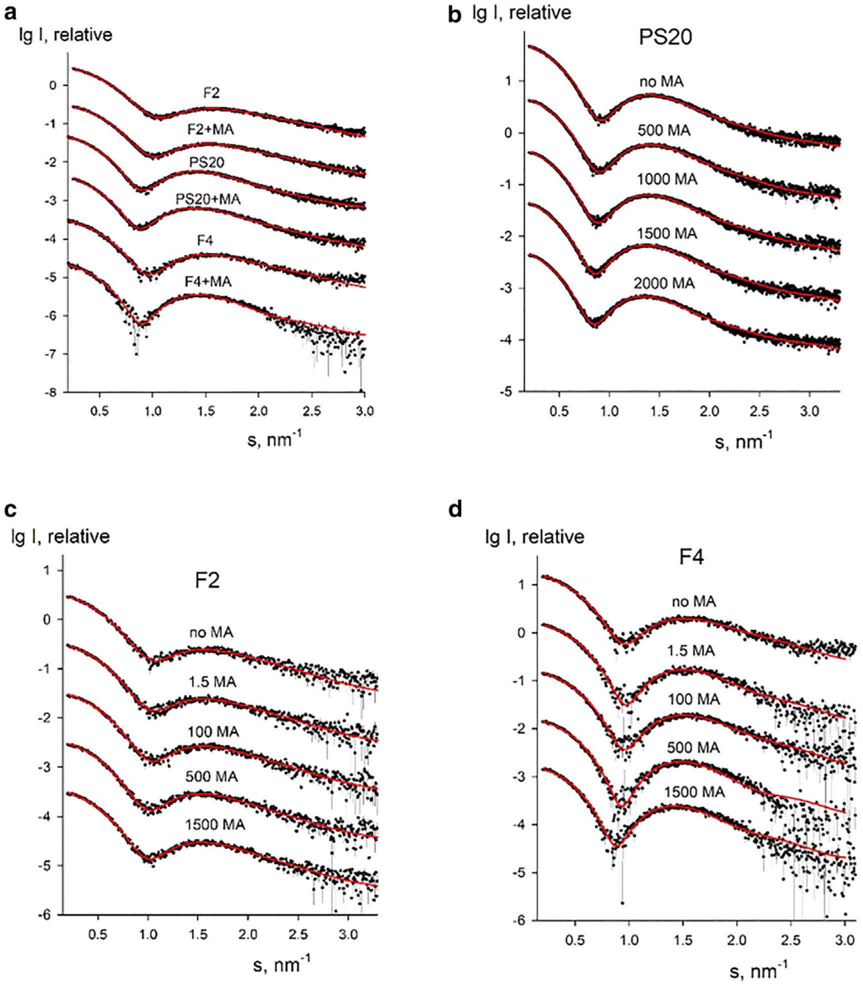

FIGURE 4 Experimental SAXS data (*dots* with error bars) and the best fits obtained by BILMIX using a model of polydisperse core-shell ellipsoidal micelles (*red curves*). (*a*) F2, PS20, and F4 with no or low amount of MA (<100 μg/mL) and with high amount of MA ("+MA") (>500 μg/mL), (*b*) PS20 in the absence of MA and with addition of MA (500, 1000, 1500, and 2000 μg/mL), (*c*) F2 in the absence of MA and with addition of MA (1.5, 100, 500, and 1500 μg/mL). (*d*) F4 in the absence of MA and with addition of MA (1.5, 100, 500, and 1500 μg/mL). For F2, PS20, and F4, the experimental data taken at 5 mg/mL are presented. To see this figure in color, go online.

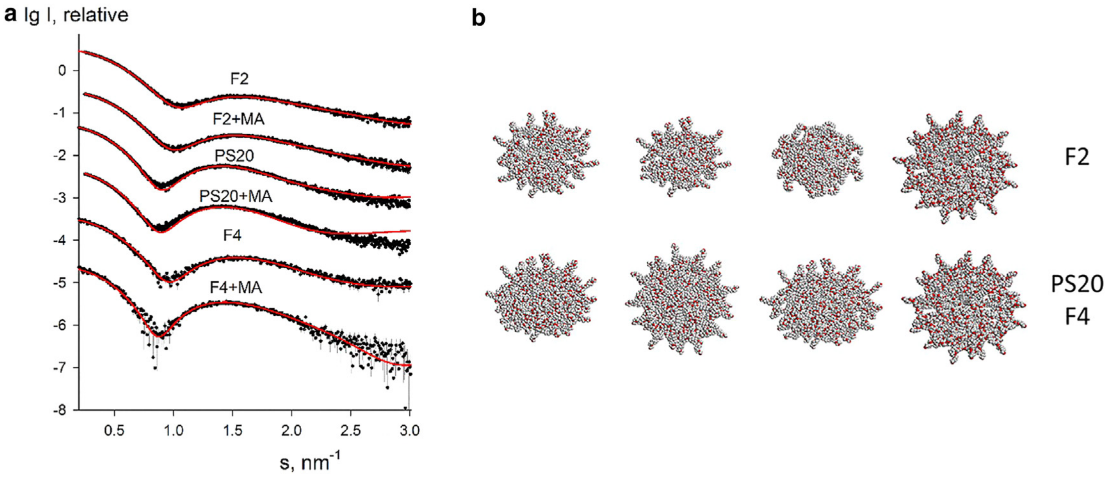

FIGURE 5 (*a*) Experimental SAXS data (*dots* with error bars) and the best fits obtained by OLIGOMER using a combination of quasiatomistic models of micelles (*red curves*). F2, PS20, and F4 with no MA and with high amount of MA ("+MA") (>500 μg/mL). (*b*) Typical quasiatomistic models of micelles selected by OLIGOMER, micelles in the upper row correspond to F2 (from left to right: model 1 [1-c18], model 5 [1-c18], model 5 [2-c12], all models contain 22 surfactant molecules, and model 6 [1-c18] with 35 molecules), micelles in the lower row are found for PS20 and F4 (from left to right: model 1 [1-c18], model 2 [1-c18], model 4 [1-c18], model 6 [1-c18], all models contain 35 surfactant molecules). Carbon and hydrogen atoms are shown as gray beads, oxygen atoms as red beads. To see this figure in color, go online.

to the SAXS data, but rather obtain a qualitative picture if and how the MA molecules would migrate into the micelles. We tried to use the MD models to fit the SAXS curves but we were not able to get a good agreement. However, the structure of PS20 was approximated by a single model and it is known that PS20 and its different fractions have no clearly defined structure (19), which renders these quantitative comparisons very challenging.

DISCUSSION

Hydrolysis of PS20 releases free fatty acids, which are poorly soluble in aqueous solutions as liquid biologics formulations. These fatty acids may precipitate into particles or droplets. Formation of these particles needs to be avoided as the absence of particles is a critical quality attribute for most liquid biologics drug products. Several investigations were conducted on this topic over the last years (2,21–30). Different factors were found to increase the fatty acid solubility in liquid formulations with PS being one of them. PS micelles appear to be an obvious reservoir for the fatty acids and this study is focused on these micelles and their interactions with free fatty acids.

PS micelles have different properties dependent on which PS subspecies is included. PS20, which is a highly diverse mixture of structures is compared with an all laurate PS20 monoester fraction (F2) and a higher-order fraction (F4) with multiple lauric acids. The SAXS analysis reveals distinct differences between these samples on their own and also upon addition of a fatty acid. Two complementary approaches using a system of polydisperse core-shell ellipsoidal micelles and an ensemble of quasiatomistic micelles gave consistent results and could well describe the data. F2 results in smaller and more polydisperse micelles than PS20 and F4. The PS20 and F4 micelles are rather similar to each other, but F4 micelles are slightly smaller and less polydisperse than PS20 micelles. Thus, the observed higher polydispersity and lower stability upon dilution of the F2 fraction can easily be explained by a lower hydrophobicity of the monoester fraction (F2) compared with the higher-order ester fraction and whole PS20. The hydrophobic core of the micelle is

TABLE 6 Volume fractions of six different quasi-atomistic models of micelles as obtained by OLIGOMER

Sample	Molecule 1-c18 (model 1)	Molecule 1-c18 (model 2)	Molecule 1-c18 (model 4)	Molecule 1-c18 (model 5)	Molecule 2-c12 (model 5)	Molecule 1-c18 (model 6)
F2 (low amount of MA)	(22 ± 2) %	–	–	(22 ± 2) %	(18 ± 1) %	(38 ± 1)%
F2 (high amount of MA)	(30 ± 2) %	–	–	(16 ± 3) %	(10 ± 2) %	(44 ± 1) %
PS20 (low amount of MA)	(31 ± 2) %	(12 ± 3) %	(57 ± 2) %	–	–	–
PS20 (high amount of MA)	–	(70 ± 2) %	(30 ± 3) %	–	–	–
F4 (low amount of MA)	(52 ± 2) %	–	–	–	–	(48 ± 3) %
F4 (high amount of MA)	(44 ± 2) %	–	(56 ± 3) %	–	–	–

The error estimates were obtained as a SD of optimized parameters from OLIGOMER runs using the angular range s_{max} (2.5 ÷ 3.0) nm^{-1} corresponding to the number of Shannon channels in the fit ($N_{shannon}$ = 5–6).

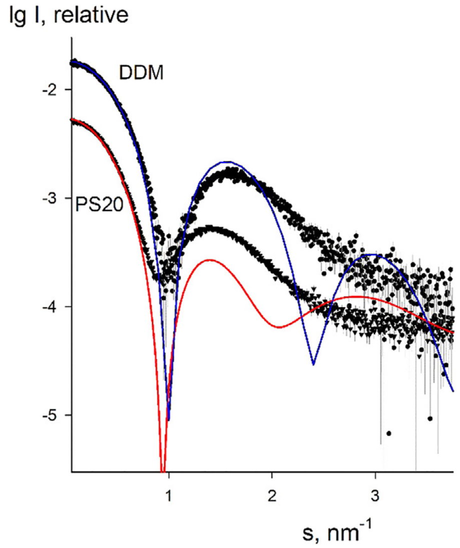

FIGURE 6 Experimental scattering from PS20 micelles at 5 mg/mL (*triangles* with error bars) and computed scattering from an ellipsoidal model constructed from 22 molecules (*red solid line*). Spheres with error bars and blue solid line are experimental scattering from DDM micelles and that from an ellipsoidal micelle with 174 molecules, respectively. To see this figure in color, go online.

the driving force for micelle formation in aqueous solutions and increases their stability (13). The effect of the hydrophobic core is less pronounced in the monoester fraction F2 than in the higher-order ester fraction F4 or whole PS20. SAXS data can be fitted by polydisperse mixtures of ellipsoidal micelles, with about from 22 to 35 molecules per micelle for F2 and 35 molecules per micelle for PS20 and F4. The addition of the fatty acid MA in concentrations up to 100 μg/mL MA reveals only marginal effects on the scattering data, within errors estimates of the micelle sizes. At the same time, for all samples, addition of high amounts of MA (>500 μg/mL) somewhat increases the average sizes (volume and shape) of the micelles indicating that MA penetrates into the surfactant micelles. As an interesting note, the electron density of MA (324 e/nm^3) is very close to that of pure water (334 e/nm^3) and the MA molecule as such has nearly zero contrast in an aqueous solution and is thus practically unseen by SAXS. The presence of MA is only revealed by the change of the size/conformation of the surfactant micelles. MD simulations of F2-like micelles (*monolauryl PS,* w = x =y = z = *5*) with different MA mixtures were performed to investigate if and how MA migrates into the F2 micelles. All simulations suggest that MA integrates into the core of the F2 micelles and indeed slightly increases their size. The individual MA molecules stack with the hydrophobic tails of the F2 molecules and their negatively charged headgroup is located at the hydrophilic outer shell of the micelles, which can be explained by the hydrophobic effect. Interestingly, the overall size of the micelles from MD simulations is smaller than the experimentally determined parameters of the micelles. There are several possible reasons for this discrepancy. The PS20 molecules are described by a single model in the simulation, which is a significant approximation given its high polydiversity. Furthermore, the GAFF force field used for the PS20 parameters is a generalizable force field and has not been specifically parametrized for micelles.

Surprisingly, smaller amounts of MA (<100 μg/mL) may be added to PS micelles solutions without noticeable effect while a measurable increase in the micelle sizes is only observed above a certain MA amount. Notably, MA is only poorly soluble in water (pH- and surfactant concentration-dependent $\leq$3 μg/mL (35)). MA amounts above the solubility limit have only limited time to interact with PS and become incorporated into the micelles before they precipitate when added to an aqueous solution. The MA amounts were added at once after being dissolved in methanol and then added to the PS solutions in our experiments. In contrast, fatty acid release into biologics upon PS hydrolysis proceeds in a rather slow and continuous manner, allowing a successive incorporation of MA into PS micelles before the MA solubility limit is reached. We assume that, in our short-term experiments, the amounts of MA added at once must be high to ensure incorporation of significant amounts of MA into the PS micelles before MA precipitates.

The experimental results and modeling indicate that fatty acids do intercalate into the hydrophobic core of PS20 micelles in aqueous solutions. Previous studies have already

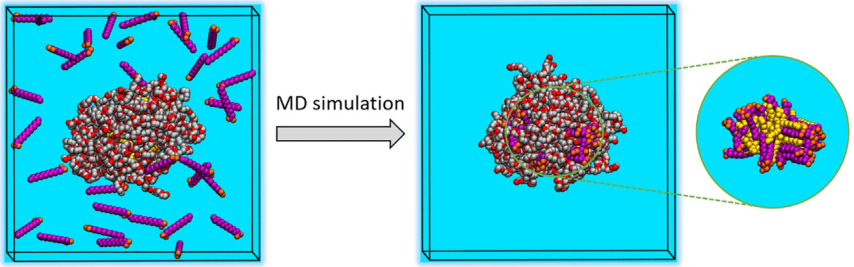

FIGURE 7 Snapshots from an MD simulation of a solvated F2 micelle (monolauryl PS with w = x = y = z = 5) with 35 F2 molecules and the addition of 35 MA molecules (1:1 mixture) at the start of the simulation (*left*) and after 300 ns (*right*). The inset shows the stacking between the hydrophobic tails of F2 and MA (F2 headgroups are not shown). Hydrophobic tails and polar headgroups of F2 molecules are shown as yellow spheres and gray/red spheres, respectively. Hydrophobic tails and polar headgroups of MA are shown as magenta and orange spheres, respectively. Hydrogens and water molecules are not shown for visual clarity. To see this figure in color, go online.

described and quantified a positive effect of PS20 on the solubility of fatty acids (35). Our results further deepen the understanding of this effect by explaining why the release of fatty acids does not necessarily result in particles and what in detail happens in the drug product. Although these observations were made in the absence of further formulation components, we expect the same mechanism to occur in liquid drug products. Finally, our results may support the planning of further investigations with the aim to better understand particle formation in biologics formulations caused by PS degradation. Open questions of relevance are, when and under which circumstances do the first particles occur and what are the possible requirements to prevent particle formation over the time period corresponding to the shelf life of the drug. More precise models of the processes could help one to predict which PS degradation and fatty acid release is acceptable and which is not.

DATA AVAILABILITY

The SAXS data were deposited in the SASBDB database (56), accession codes: SASDRN9, SASDRQ9, and SASDRS9 for PS20, F2, and F4 without MA or with low amount of MA ($<$100 μg/mL), respectively; SASDRP9, SASDRR9, and SASDRT9 for PS20, F2, and F4 with high amount of MA) ($>$500 μg/mL), respectively.

SUPPORTING MATERIAL

Supporting material can be found online at https://doi.org/10.1016/j.bpj.2023.06.011.

AUTHOR CONTRIBUTIONS

Conceptualization, J.E., D.I.S., and N.N.; methodology, J.E., T.W.G., and P.V.K.; investigation, J.E. and T.W.G.; writing – original draft, J.E., T.W.G., and H.G.; writing – review & editing, J.E., T.W.G., H.G., D.I.S., and N.N.; formal analysis, J.E., T.W.G., H.G., and P.V.K.; molecular dynamics calculations, H.G.; data evaluation, P.V.K.; supervision, D.I.S. and N.N.; project administration, N.N.

ACKNOWLEDGMENTS

The authors gratefully acknowledge the company Croda for providing the PS fractions F2 and F4, Martin Huelsmeyer from AbbVie for establishing the Croda cooperation, and Friederike Junge from AbbVie for initiating this study.

P.V.K. acknowledges support from Ministry of Science and Higher Education of the Russian Federation within the State assignment FSRC "Crystallography and Photonics" RAS (SAXS analysis).

DECLARATION OF INTERESTS

J.E., H.G., and N.N. are employees of AbbVie. The study design and financial support for this study were provided by AbbVie. AbbVie participated in the interpretation of data, review, and approval of the publication.

REFERENCES

1. Li, S., C. Schöneich, and R. T. Borchardt. 1995. Chemical instability of protein pharmaceuticals: Mechanisms of oxidation and strategies for stabilization. *Biotechnol. Bioeng.* 48:490–500.
2. Li, J., M. E. Krause, …, L. Zhu. 2019. Interfacial Stress in the Development of Biologics: Fundamental Understanding, Current Practice, and Future Perspective. *AAPS J.* 21:44.
3. Patro, S. Y., E. Freund, and B. S. Chang. 2002. Protein formulation and fill-finish operations. *Biotechnol. Annu. Rev.* 8:55–84.
4. Wuchner, K., L. Yi, …, V. Le Brun. 2022. Industry perspective on the use and characterization of polysorbates for biopharmaceutical products Part 1: Survey report on current state and common practices for handling and control of polysorbates. *J. Pharm. Sci.* 111:1280–1291.
5. Joshi, O., L. Chu, …, D. Q. Wang. 2009. Adsorption and function of recombinant Factor VIII at the air–water interface in the presence of Tween 80. *J. Pharm. Sci.* 98:3099–3107.
6. Kapp, S. J., I. Larsson, …, L. Jorgensen. 2015. Competitive Adsorption of Monoclonal Antibodies and Nonionic Surfactants at Solid Hydrophobic Surfaces. *J. Pharm. Sci.* 104:593–601.
7. Agarkhed, M., C. O'Dell, …, A. Srivastava. 2018. Effect of Surfactants on Mechanical, Thermal, and Photostability of a Monoclonal Antibody. *AAPS PharmSciTech.* 19:79–92.
8. Bam, N. B., J. L. Cleland, …, T. W. Randolph. 1998. Tween protects recombinant human growth hormone against agitation-induced damage via hydrophobic interactions. *J. Pharm. Sci.* 87:1554–1559.
9. Liu, L., W. Qi, …, J. F. Carpenter. 2013. The effects of excipients on protein aggregation during agitation: An interfacial shear rheology study. *J. Pharm. Sci.* 102:2460–2470.
10. Grigoriev, D., S. Derkatch, …, R. Miller. 2007. Relationship between structure and rheological properties of mixed BSA/Tween 80 adsorption layers at the air/water interface. *Food Hydrocolloids.* 21:823–830.
11. Lee, H. J., A. McAuley, …, J. McGuire. 2011. Molecular origins of surfactant-mediated stabilization of protein drugs. *Adv. Drug Deliv. Rev.* 63:1160–1171.
12. Khan, T. A., H. C. Mahler, and R. S. K. Kishore. 2015. Key interactions of surfactants in therapeutic protein formulations: A review. *Eur. J. Pharm. Biopharm.* 97:60–67.
13. Maibaum, L., A. R. Dinner, and D. Chandler. 2004. Micelle Formation and the Hydrophobic Effect. *J. Phys. Chem. B.* 108:6778–6781.
14. Nagarajan, R., and E. Ruckenstein. 1991. Theory of surfactant self-assembly: a predictive molecular thermodynamic approach. *Langmuir.* 7:2934–2969.
15. Stephenson, B. C., K. Beers, and D. Blankschtein. 2006. Complementary Use of Simulations and Molecular-Thermodynamic Theory to Model Micellization. *Langmuir.* 22:1500–1513.
16. Tanford, C. 1974. Theory of micelle formation in aqueous solutions. *J. Phys. Chem.* 78:2469–2479.
17. Rosen, M., and J. T. Kunjappu. 2012. Surfactants and Interfacial Phenomena. John Wiley & Sons, Inc.
18. Strickley, R. G., and W. J. Lambert. 2021. A review of Formulations of Commercially Available Antibodies. *J. Pharm. Sci.* 110:2590–2608.e56.
19. Fekete, S., K. Ganzler, and J. Fekete. 2010. Fast and sensitive determination of Polysorbate 80 in solutions containing proteins. *J. Pharm. Biomed. Anal.* 52:672–679.
20. Knoch, H., M. H. Ulbrich, …, H. Heerklotz. 2021. Complex Micellization Behavior of the Polysorbates Tween 20 and Tween 80. *Mol. Pharm.* 18:3147–3157.
21. Labrenz, S. R. 2014. Ester hydrolysis of polysorbate 80 in mAb drug product: evidence in support of the hypothesized risk after the observation of visible particulate in mAb formulations. *J. Pharm. Sci.* 103:2268–2277.
22. Dixit, N., N. Salamat-Miller, …, S. K. Basu. 2016. Residual Host Cell Protein Promotes Polysorbate 20 Degradation in a Sulfatase Drug

Product Leading to Free Fatty Acid Particles. *J. Pharm. Sci.* 105: 1657–1666.

23. Chiu, J., K. N. Valente, …, K. H. Lee. 2017. Knockout of a difficult-to-remove CHO host cell protein, lipoprotein lipase, for improved polysorbate stability in monoclonal antibody formulations. *Biotechnol. Bioeng.* 114:1006–1015.
24. Dwivedi, M., M. Blech, …, P. Garidel. 2018. Polysorbate degradation in biotherapeutic formulations: Identification and discussion of current root causes. *Int. J. Pharm.* 552:422–436.
25. Tomlinson, A., B. Demeule, …, S. Yadav. 2015. Polysorbate 20 degradation in biopharmaceutical formulations: quantification of free fatty acids, characterization of particulates, and insights into the degradation mechanism. *Mol. Pharm.* 12:3805–3815.
26. Roy, I., A. Patel, …, K. Amin. 2021. Polysorbate degradation and particle formation in a high concentration mAb: formulation strategies to minimize effect of enzymatic polysorbate degradation. *J. Pharm. Sci.* 110:3313–3323.
27. Doshi, N., J. Giddings, …, M. Hu. 2021. A Comprehensive Assessment of All-Oleate Polysorbate 80: Free Fatty Acid Particle Formation, Interfacial Protection and Oxidative Degradation. *Pharm. Res. (N. Y.).* 38:531–548.
28. Lei, M., C. Quan, …, C. Schöneich. 2021. Light-Induced Histidine Adducts to an IgG1 Molecule Via Oxidized Histidine Residue and the Potential Impact of Polysorbate-20 Concentration. *Pharm. Res. (N. Y.).* 38:491–501.
29. Vaclaw, C., K. Merritt, …, P. Dhar. 2021. Impact of polysorbate 80 grade on the interfacial properties and interfacial stress induced subvisible particle formation in monoclonal antibodies. *J. Pharm. Sci.* 110:746–759.
30. Allmendinger, A., V. Lebouc, …, K. Abstiens. 2021. Glass leachables as a nucleation factor for free fatty acid particle formation in biopharmaceutical formulations. *J. Pharm. Sci.* 110:785–795.
31. Ha, E., W. Wang, and Y. J. Wang. 2002. Peroxide formation in polysorbate 80 and protein stability. *J. Pharm. Sci.* 91:2252–2264.
32. Doshi, N., K. Rutherford, and A. Najjar. 2021. Dissolution of Polysorbate 20 degradation related free fatty acid particles in intravenous bag solutions. *J. Pharm. Sci.* 110:687–692.
33. Doshi, N., J. Martin, and A. Tomlinson. 2020. Improving Prediction of Free Fatty Acid Particle Formation in Biopharmaceutical Drug Products: Incorporating Ester Distribution during Polysorbate 20 Degradation. *Mol. Pharm.* 17:4354–4363.
34. Fish, R., J. Lin, and N. Doshi. 2020. Impact of Silicone Oil on Free Fatty Acid Particle Formation due to Polysorbate 20 Degradation. *Pharm. Res. (N. Y.).* 37:216.
35. Doshi, N., B. Demeule, and S. Yadav. 2015. Understanding Particle Formation: Solubility of Free Fatty Acids as Polysorbate 20 Degradation Byproducts in Therapeutic Monoclonal Antibody Formulations. *Mol. Pharm.* 12:3792–3804.
36. Lapelosa, M., T. W. Patapoff, and I. E. Zarraga. 2016. Molecular simulations of micellar aggregation of polysorbate 20 ester fractions and their interaction with N-phenyl-1-naphthylamine dye. *Biophys. Chem.* 213:17–24.
37. Blanchet, C. E., A. Spilotros, …, D. I. Svergun. 2015. Versatile sample environments and automation for biological solution X-ray scattering experiments at the P12 beamline (PETRA III, DESY). *J. Appl. Crystallogr.* 48:431–443.
38. Round, A., F. Felisaz, …, F. Cipriani. 2015. BioSAXS Sample Changer: a robotic sample changer for rapid and reliable high-throughput X-ray solution scattering experiments. *Acta Crystallogr. D Biol. Crystallogr.* 71:67–75.
39. Hajizadeh, N. R., D. Franke, and D. I. Svergun. 2018. Integrated beamline control and data acquisition for small-angle X-ray scattering at the P12 BioSAXS beamline at PETRAIII storage ring DESY. *J. Synchrotron Radiat.* 25:906–914.
40. Franke, D., A. G. Kikhney, and D. I. Svergun. 2012. Automated acquisition and analysis of small angle X-ray scattering data. *Nucl Instruments Methods Phys Res Sect Accel Spectrometers Detect Assoc Equip.* 689:52–59.
41. Konarev, P. V., V. V. Volkov, …, D. I. Svergun. 2003. PRIMUS: a Windows PC-based system for small-angle scattering data analysis. *J. Appl. Crystallogr.* 36:1277–1282.
42. Svergun, D. I. 1992. Determination of the regularization parameter in indirect-transform methods using perceptual criteria. *J. Appl. Crystallogr.* 25:495–503.
43. Konarev, P. V., M. V. Petoukhov, …, E. V. Shtykova. 2020. BILMIX: a new approach to restore the size polydispersity and electron density profiles of lipid bilayers from liposomes using small-angle X-ray scattering data. *J. Appl. Crystallogr.* 53:236–243.
44. Manalastas-Cantos, K., P. V. Konarev, …, D. Franke. 2021. ATSAS 3.0: expanded functionality and new tools for small-angle scattering data analysis. *J. Appl. Crystallogr.* 54:343–355.
45. Svergun, D., C. Barberato, and M. H. J. Koch. 1995. CRYSOL – a Program to Evaluate X-ray Solution Scattering of Biological Macromolecules from Atomic Coordinates. *J. Appl. Crystallogr.* 28:768–773.
46. Abraham, M. J., T. Murtola, …, E. Lindahl. 2015. GROMACS: High performance molecular simulations through multi-level parallelism from laptops to supercomputers. *SoftwareX.* 1-2:19–25.
47. Wang, J., R. M. Wolf, …, D. A. Case. 2004. Development and testing of a general amber force field. *J. Comput. Chem.* 25:1157–1174.
48. Darden, T., D. York, and L. Pedersen. 1993. Particle mesh Ewald: An N ·log(N) method for Ewald sums in large systems. *J. Chem. Phys.* 98:10089–10092.
49. Miyamoto, S., and P. A. Kollman. 1992. Settle: An analytical version of the SHAKE and RATTLE algorithm for rigid water models. *J. Comput. Chem.* 13:952–962.
50. Hess, B. 2008. P-LINCS: A Parallel Linear Constraint Solver for Molecular Simulation. *J. Chem. Theory Comput.* 4:116–122.
51. Hopkins, C. W., S. Le Grand, …, A. E. Roitberg. 2015. Long-Time-Step Molecular Dynamics through Hydrogen Mass Repartitioning. *J. Chem. Theory Comput.* 11:1864–1874.
52. Bussi, G., D. Donadio, and M. Parrinello. 2007. Canonical sampling through velocity rescaling. *J. Chem. Phys.* 126, 014101.
53. Nayem, J., Z. Zhang, …, Y. Liu. 2020. Micellar Morphology of Polysorbate 20 and 80 and Their Ester Fractions in Solution via Small-Angle Neutron Scattering. *J. Pharm. Sci.* 109:1498–1508.
54. Gabel, F., S. Engilberge, …, E. Girard. 2019. Medical contrast media as possible tools for SAXS contrast variation. *Iucrj.* 6:521–525.
55. Ivanović, M. T., M. R. Hermann, …, J. S. Hub. 2020. Small-Angle X-ray Scattering Curves of Detergent Micelles: Effects of Asymmetry, Shape Fluctuations, Disorder, and Atomic Details. *J. Phys. Chem. Lett.* 11:945–951.
56. Kikhney, A. G., C. R. Borges, …, D. I. Svergun. 2020. SASBDB: Towards an automatically curated and validated repository for biological scattering data. *Protein Sci.* 29:66–75.

Biophysical Journal, Volume *122*

Supplemental information

Small-angle x-ray scattering investigation of the integration of free fatty acids in polysorbate 20 micelles

Jörg Ehrit, Tobias W. Gräwert, Hendrik Göddeke, Petr V. Konarev, Dmitri I. Svergun, and Norbert Nagel

Supplementary Material

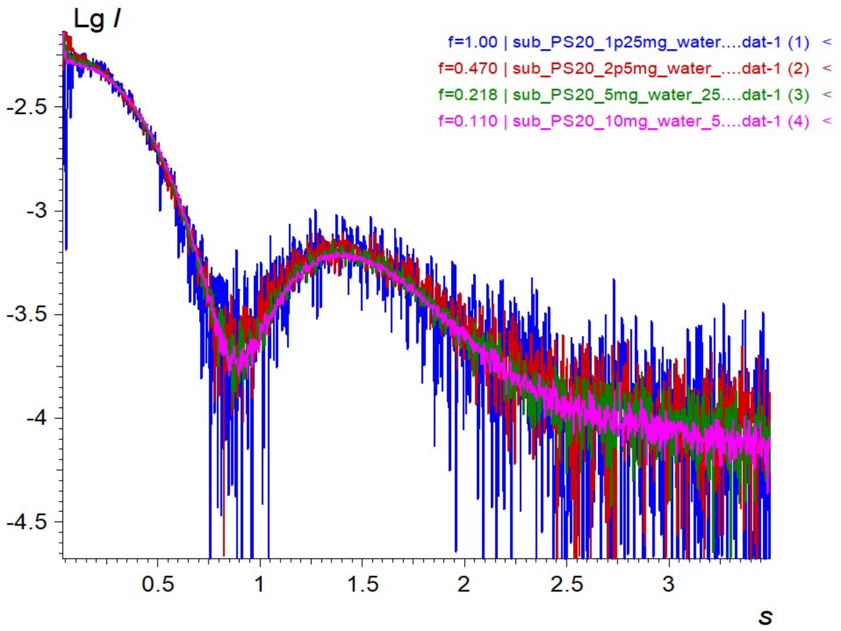

Figure S1. Scattering from PS20 at concentrations 1.25 mg/ml (curve 1), 2.5 mg/ml (curve 2), 5.0 mg/ml (curve 3) and 10.0 mg/ml (curve 4). The s-axis units are nm^{-1}.

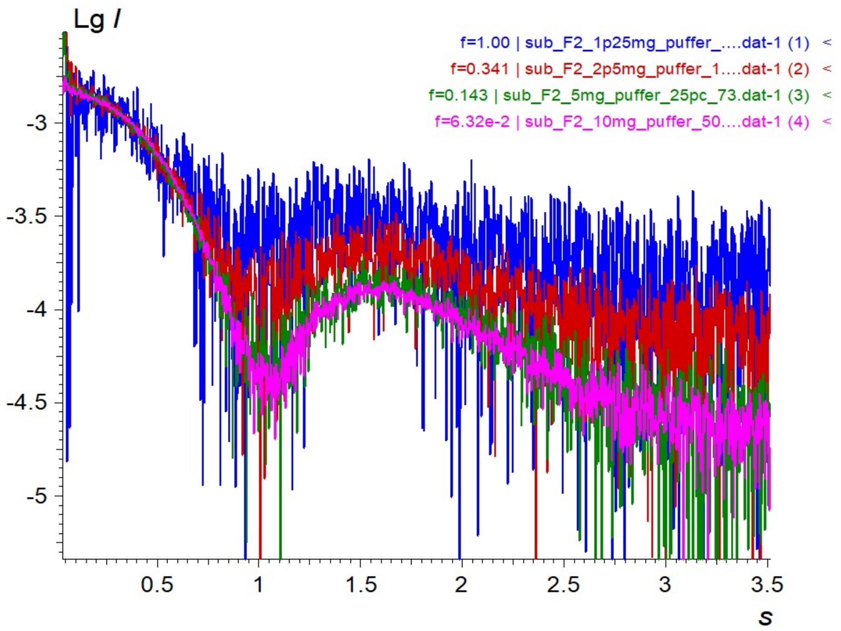

Figure S2. Scattering from fraction F2 at concentrations 1.25 mg/ml (curve 1), 2.5 mg/ml (curve 2), 5.0 mg/ml (curve 3) and 10.0 mg/ml (curve 4). The s-axis units are nm^{-1}.

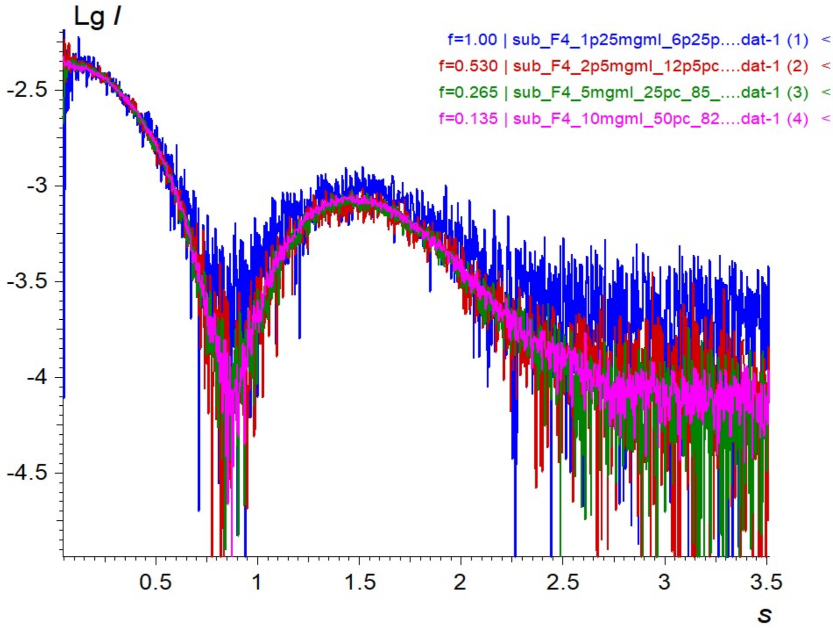

Figure S3. Scattering from fraction F4 at concentrations 1.25 mg/ml (curve 1), 2.5 mg/ml (curve 2), 5.0 mg/ml (curve 3) and 10.0 mg/ml (curve 4). The s-axis units are nm^{-1}.

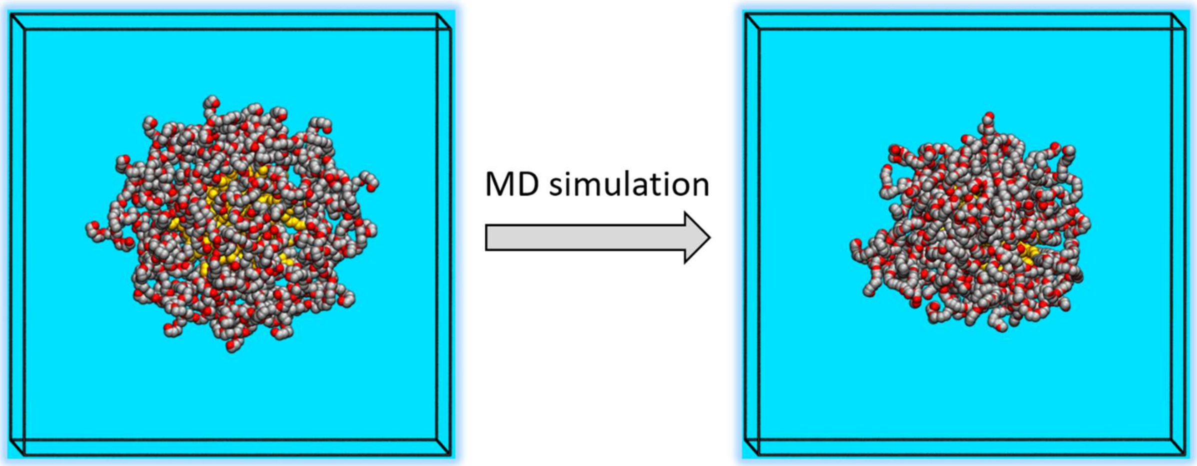

Figure S4. *Snapshots from an MD simulation of a F2 micelle model (generated by OLIGOMER, see. model 6 in Fig. 5) with 35 F2 molecules solvated with water at the start of the simulation (left) and after 300 ns (right). Hydrophobic tails and polar head groups of F2 molecules are shown as yellow spheres and grey/red spheres, respectively. Hydrogens and water molecules are not shown for visual clarity.*

MD models and force field parameters

The compressed zip file "MD-models-and-forcefield-params.zip" includes representative models of the F2 micelle at the start and at the end of the MD simulations (Fig. S4) w/o myristic acid, and at the end of the simulation upon adding myristic acid at a 1:1 mixture (see Fig. 7). All GAFF2 force field parameters of the F2 and myristic acid molecules can be found in "forcefield-params-for-F2-and-MYR.txt". The detailed simulation protocol is outlined in the Materials and Methods section.
